# Welfare technology, ethics and well-being a qualitative study about the implementation of welfare technology within areas of social services in a Swedish municipality

**DOI:** 10.1080/17482631.2020.1835138

**Published:** 2020-10-25

**Authors:** Marta Cuesta, Lena German Millberg, Staffan Karlsson, Susann Arvidsson

**Affiliations:** School of Health and Welfare, Halmstad University, Halmstad, Sweden

**Keywords:** Ethical analysis, health spaces, welfare technology, well-being, working model

## Abstract

**Purpose:** Digitalization and e-health have potential to generate good quality, equal health, well-being and to develop and strengthen individuals’ resources with the goal of increased independence and participation in society. The implementation of welfare technology requires knowledge of digitalization, as well as an awareness of its meaning in terms of ethical principles and ethical analysis. The purpose of this study was to describe ethical analysis concerning the implementation of welfare technology, in terms of both strategies and tools, within areas of social services in a Swedish municipality.

**Method:** We followed a working model that focused on increased knowledge and experience in the implementation of welfare technology from an ethical perspective. In the data collection were observations, a questionnaire with open-ended questions and focus group discussions used.

**Results:** The analysis showed that when welfare technology was introduced and implemented within the area of social services in a municipality, ethical awareness resulting from the conflicts between various interests and values had to be addressed.

**Conclusions:** The ethical analysis improved implementation of strategies and tools in terms of facts and values, and invisible underlying values to the concept of well-being.

## Background and aim

Welfare technology is a concept that has been used by policy makers over the last decade, referring to a digital transformation and system-wide approach beyond a single assistive technology, and in order to ensure that the work within health and social care is carried out with a focus on the individual´s needs. Also, welfare technology has been referred to satisfy the requirements for being able to maintain good conditions for measures related to health and well-being (Andersson et al., 2019; The National Board of Health and Welfare, 2018).

International studies remark that the implementation of welfare technology requires knowledge of digitalization (King et al., 2012; Nilsen et al., 2016; Peek et al., 2016), as well as an awareness of its meaning in terms of ethical principles and ethical analysis (Hofmann, 2012; Zwijsen et al., 2011). Such ethical awareness may contribute to an increased transparency in the interaction between staff and individuals (in this study, people with disabilities).

In Europe the health care, social care and support of the social welfare administration shall be grounded on values, norms and be provided with evidence-based approaches and interventions. In general, ethics and morals are often used synonymously, although the concepts have different meanings. Morals refer to a person’s practical and actual actions, which means that the person’s specific actions refer to morals. Ethics represent the systematic reflection on a person’s actions and the motives for such actions. Ethics can be understood as being the theory of morality in the sense that ethics analyse, interpret, examine and systematize the principles that can be used to argue for and against a certain action in a situation, while the morals of a person or group are shown through actions (European Group on Ethics in Science and New Technologies [EGE], 2018; The Swedish National Council on Medical Ethics, 2018). A number of ethical principles govern moral behaviour. The principle of human dignity promotes the equal value of all people, which means that all people have the same human rights and the same right to be respected (Beauchamp & Childress, 2009). The principle of autonomy means that each person has the right to make decisions regarding their own life, but not in a way that infringes on the autonomy of others. In order to be able to make decisions and exercise autonomy, a person needs access to accurate information and possess the competence to be able to make appropriate decisions. The principle of benefit must govern the work and interpersonal treatment within health and social care in order to promote health and well-being in individuals with disabilities. The principle of doing no harm means avoiding unjustified risks. The goal of health and social care shall be to minimize injury and prevent suffering in people with disabilities (Beauchamp & Childress, 2009). The principle of justice is based on that the one who needs the most gets the most (Rawls, 2005).

The implementation of welfare technology in municipalities may lead to organizational, cultural, technical and ethical resistance among staff (Andersson et al., 2016). Organizationally, this may involve a resistance to changes in established procedures, necessary skills development and communication between groups and professions. From a cultural perspective, this may, for example, entail resistance to linguistic differences, but may also involve a clash between different professional cultures. Technically, it may entail resistance to the technology itself, in regard to function and safety (Novitzky et al., 2015). Ethical resistance may be based on issues related to the safety and quality of care (Coughlin, 2010), as well as the integrity of individuals with disabilities (Fischer et al., 2014; Landau et al., 2010; Niemeijer et al., 2010). The various forms of resistance appear to stem from a feeling of being threatened and generates a fear of change, of losing power or control, as well as losing moral or professional integrity (Nilsen et al., 2016). There may also be feelings of alienation when advanced technology is used in the home. There may be conflicting goals but also difficulties in respecting the integrity, dignity and vulnerability of people with disabilities (Fischer et al., 2014; Landau et al., 2010; Niemeijer et al., 2010) and in guaranteeing equal access and a fair distribution of welfare technology (Hofmann, 2012). As well as various forms of resistance and challenges, there are also benefits to implementing welfare technology in the form of increased safety and independence, a sense of usability and a reduced burden for relatives in terms of well-being (Peek et al., 2014).

Studies have shown that welfare technology can give individuals with disabilities increased independence and freedom in their daily lives (Landau et al., 2010; Melander Wikman et al., 2008; Niemeijer et al., 2010; Werner & Landau, 2011; Zwijsen et al., 2011). Welfare technology has been described as enabling people to remain longer in their own homes and independently manage their daily lives, which contributes to the well-being of individuals with disabilities (Essén, 2008; Novitzky et al., 2015). It has also been proposed that opportunities for individuals with disabilities to decide for themselves whether and how welfare technology is to be used is crucial to the success of welfare technology solutions in increasing a person’s well-being (Coughlin, 2010; Melander Wikman et al., 2008; Wilkowska & Ziefle, 2012).

Welfare technology constitutes a part of different social contexts in Sweden as well as in the Nordic countries and beyond. The essence and goal of introducing welfare technology is to increase the quality of life and the well-being of people in various societal spaces (Andersson et al., 2019). Also, as this study addresses, it involves contributing more specifically to increased collaboration between different areas of social welfare administration and providing support to people with disabilities. Welfare technology can therefore be understood to be an integrated part of the health space, and as a tool for achieving the vision for e-health within the framework of health and social care (Andersson et al., 2019; The National Board of Health and Welfare, 2018).

The importance of ethical analysis is to contribute to increased awareness regarding aspects associated with invisible values (The Swedish National Council on Medical Ethics, 2018). Ethical analysis can be understood to be a process constituting several aspects. Firstly, the implementation of welfare technology may have moral consequences. Secondly, welfare technology brings values with it and may challenge the prevailing values in society that should be addressed. Thirdly, a more fundamental insight is required into the way in which welfare technology as a whole can improve health care and well-being. (Samuli et al., 2008). Thus, **the aim** of this study was to describe ethical analysis concerning the implementation of welfare technology, in terms of both strategies and tools, within areas of social services in a Swedish municipality.

## Method and material

This study is based on a project that originated within the framework of a collaborative project called “HiCube—competent healthcare” from September 2015–August 2018, funded by the European Social Fund (ESF), Halmstad University and the Region of Halland in Sweden. The aim of the “Welfare Technology from an Ethical Perspective” project was to create conditions for the increased use of welfare technology by highlighting opportunities and challenges based on ethical aspects regarding the implementation and use of welfare technology within areas of social services in a municipality.

### Design

A qualitative design was used in this study entailing a systematic subjective approach to describing experiences with the aim of gaining insight and explaining the depth and complexity of a phenomenon (Polit & Tatano Beck, 2018).

### The working model

**Figure 1.**
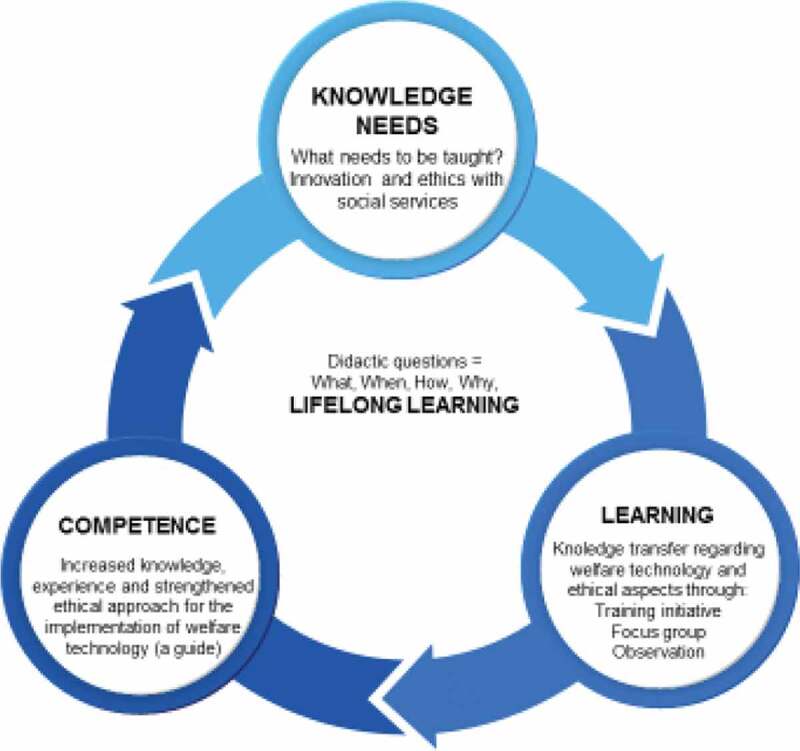
The working model for increased competence in the implementation of welfare technology concerning ethics

The working model was based on scientific knowledge and proven experience. The working model (Figure 1) was created to develop a learning process regarding strategies for increased knowledge and experiences based on awareness to ethical analysis associated with the implementation of welfare technology. In other words, that there exist needs of new knowledge by learning and competence. Learning is about changing our way of understanding our surroundings, but also represents a lasting change in an individual’s competence through interaction with the environment (experiential exchange) (Bowden & Marton, 1998; Ellström, 1992). Learning continues throughout an individual’s life and is an ongoing process, so-called lifelong learning (Marton & Booth, 2000).

The working model involved parallel processes: two observations in two residential homes that offered special services; training days followed by a questionnaire with open-ended questions; and focus group discussions. The whole working model programme was based on awareness of ethical analysis associated with the implementation of welfare technology.

The working model started with training days for participants who were employed within the social welfare administration of a municipality in southern Sweden. All departments were invited. The managers relayed the relevant information and approved participation during working hours. The participants attended one training day each. The training days covered four different areas of knowledge. The knowledge area “Law and ethics” highlighted how technology and law in connection with ethical dilemmas can be managed. The knowledge area “Health innovation” provided reflections on health innovation and digitalization through service design. Within the knowledge area “Collaboration and test environments”, the Centre for Health Technology Halland (HCH) presented various services such as health innovation, Test Environment Halland and cluster initiatives. The knowledge area “Norm criticism and gender equality” highlighted aspects of welfare technology based on critical thinking, as well as the future of health and social care with the home as a base. Each training day also included a questionnaire with open-ended questions, and focus group discussion, with the ambition to introduce attention to the need for knowledge about the needs of more competence based on ethical analysis associated with the implementation of welfare technology.

#### Observations

The residential home housed for people with disabilities who were in need of support and service around the clock. The residential home consisted of eight apartments, but there were also common areas. Two staff members and eight people with disabilities at the residential home participated in the observations. The daily activity centre comprised 40 people with disabilities and 18 staff, of which 16 staff and eight people with disabilities were involved in the observations.

The observations (Merriam, 1994) involved one researcher and one expert in welfare technology and digitalization visiting the facilities in question. The observations began with a study visit to each facility, where the study was presented to the staff. The staff could then ask questions and the researcher and expert became acquainted with the activities concerning ethics. The study also included conversations with unit managers and employees, which took place during a workplace meeting at each facility. Conversations with people with disabilities were limited to the planned visits for observations in each facility. In other words, these conversations would not be included as data in the analysis.

Ethical principles regarding how the research was conducted were explained and consent forms were distributed. An observation guide was then developed that focused on the need for welfare technology within activities, and how, where and when welfare technology was used. Furthermore, the observation guide addressed how welfare technology could support the quality of life of people with disabilities, how welfare technology facilitates and supports the work of staff, as well as the potential development areas for welfare technology.

#### The questionnaire

During the training days the participants (n = 106) were asked to answer a questionnaire with open-ended questions about welfare technology within health and social care and socio-demographic issues. Of the participants, 67 answered the questionnaire. The open questions were as follows: What does welfare technology mean to you? What ethical issues do you face in your work when it comes to welfare technology? The majority of the participants were female (87%) with an average age of 46 (27–62 years) with a few born outside of Sweden (9%). Most had a university level education (97%) and had extensive experience in their profession (average 9 years) and duration of working at their current workplace (average 7.5 years).

#### Focus group discussions

Of the 106 employees who participated in the training days, 33 participated in the focus group discussions, 28 female (85%) and five males with an average age of 45 (27–63 years). In the selection, variation was sought in each focus group with regard to age, gender, education, country of birth, profession, number of years in the profession and current work. In total, seven focus groups were held with 4–5 people in each focus group. Focus group discussions were based on a qualitative research method in which a group of people engage in discussion on a certain theme or issue in order to gain knowledge about the group’s ideas, attitudes and values (Wibeck, 2010). The participants mainly comprised unit managers, method developers, social workers, support educators and development managers who, on average, had worked 7.6 years at their current workplace.

The focus group discussions were held after the lectures during the training days. The invitations indicated that participation was voluntary and that participants could discontinue at any time without providing any explanation. During the focus group discussions, an interview guide was used with questions concerning opportunities and challenges, as well as ethical aspects relating to the implementation and use of welfare technology in organizations. During the focus group discussions, one researcher served as a moderator and another researcher served as an observer. The focus group discussions were recorded and then transcribed verbatim and analysed.

### Data analysis

The different parts of the working model helped to situate the collected data within the context of the whole narrative. The data analysis was conducted using a narrative analysis (Hyden & Hyden, 1997). The material was transcribed and read several times by the researchers and has provided interdisciplinary depth to the entire study. The researchers initially analysed the data separately and then compared their findings. The data analysis followed several steps. The first step was to identify the interconnection between themes from each data collection. The second step was to present the results from each data collection with respect to the themes in focus in order to strengthen the confirmability of the interlinked arguments to be raised in the final discussion. To further strengthen confirmability, the researchers considered and critically reflected on their own preunderstanding. The third step was to use the final discussion to address the key questions of the study.

#### Ethical aspects

The entire study was conducted with the approval of the Regional Ethical Review Board in Lund (no.: 2017/578). The participants were informed both verbally and in writing about the study and that their participation was voluntary. They were assured of confidentiality and their right to withdraw at any time without having to justify their decision. Following data collection, the participants were given an opportunity to discuss any feelings or thoughts they had.

## Findings

The findings are structured in three themes: *Welfare technology in health spaces*, highlighting the results of the observations; *Ethics and spaces*, illuminating the results of the questionnaire and *Healthcare activities and ethical values*, presenting the focus group discussions; particularly focusing on narratives about the impact of the ethical analysis in the organizations involved in the study.

### Welfare technology in health spaces

Each person with disabilities had a “contact person” in the staff group with overall responsibility for support and service measures. The regular staff were responsible for planned activities adapted to the needs of people and their disabilities, which were documented in an implementation plan. The documentation of information and communication in collaboration took place via notes in paper form and telephone calls.

#### Residential home with special services

All the people with disabilities lived at Höghuset a residential home, in their own apartment. The building was relatively new and equipped with advanced technology (e.g., IT sockets). The building also had an apartment that served as a base for the staff during their work shifts, and which was open to people visiting the residents. Everyone indicated that they were happy there. The technical aids in the apartments varied depending on the occupant. The view from Höghuset was very relaxing. The sea was close by and the scenic surroundings encouraged the residents to take relaxing strolls. The staff talked about peaceful outings, but also about their concern when people with disabilities did not return home on time. “Where are they?” the staff would wonder. They explained that “we would wait for a while and then call the individual’s phone”, even though their actions might be perceived as controlling. The people with disabilities appreciated the support of the staff, but emphasized the importance of the staff respecting their independence, which is in line with the ethical principle of autonomy.

The support measures were described and documented for each person with disabilities in a so-called method file. Morning tasks consisted of waking the residents, helping with breakfast, contacting the residents’ daily activity centres in order to coordinate activities, as well as calling for taxis so that the resident can come to the residential home. The afternoon tasks consisted of support for cleaning, cooking, laundry, writing shopping lists, accompanying residents during activities such as bicycle trips, contact at bedtime, etc. All documentation and information regarding support and service measures was managed via analogue methods (i.e., method files and message boards) in which activities were updated on a daily basis. Communication with other organizations such as daily activity centres and taxi companies, as well as selected conversations with residents, took place via phone. All these activities were carried out in a normal way, but without any deeper awareness of the impact of ethical principles.

The welfare technology that was installed was managed by the staff and the people with disabilities. The technology was primarily used to help with basic needs. The staff followed an implementation plan and provided support to the residents via visits to their apartments and via telephone contact. The residents managed their day-to-day lives with the help of the staff, although they stated that more advanced technology would have helped them. Both management and staff showed an awareness of the need and benefits of more advanced welfare technology associated with support activities. The staff highlighted the notice board (whiteboard) and indicated that many residents used this function, i.e., knocked on the door and checked in and out on the board on their way to and from their respective activities. However, the residents adopted this routine because they wanted to come in and talk to the staff, which they described as cheering them up.

In the staff apartment, a TV monitor was used for resident-related purposes (e.g., watching a show). The staff suggested that the “TV monitor could be used more within the context of their work (e.g., presentations during meetings or distance education)”. The residents’ apartments had various activity calendars where staff reminded the residents and their relatives about planned daily activities. The staff indicated that “sometimes there were several calendars on display in a resident’s apartment, which created confusion for the person concerned”. The staff called for more advanced technology in order to be better equipped for cooperating with the residents and simplifying their daily lives. One member of staff suggested that “individualized technology had contributed to the personal development of the residents, for example, talking watches and more advanced features on their phones”. One of the residents spoke enthusiastically about “talking watches and that acquiring one was their main priority”. Another resident spoke about “dreaming of doing their own shopping online via a phone app”. The staff took care of the residents in an ethically-appropriate manner, but sometimes they express feelings of unsureness when advanced technology was implemented, situations which entails awareness of ethical principles.

Awareness of the importance of welfare technology was not only evident through the spontaneous conversations during observations, but also during a workplace meeting concerning ethical analysis, between the manager and staff. The staff emphasized that increased integration of advanced technology would contribute to creating a “common knowledge bank” with focus on ethics, both for staff and for people with disabilities. This would improve clarity concerning support efforts, and additional knowledge in welfare technology was also considered to stimulate the staff. Welfare technology was also described as a developmental factor for the residents, which increased their well-being.

#### Daily activity centre

The building was situated in a quiet green area on the outskirts of the city and close to the sea. The staff who accompanied us during the observation tour told us that the building had been renovated fairly recently. One member of staff pointed out that “there had been a vision for how advanced technology would be integrated into the building from the very start”. A deeper awareness of what this kind of technology entailed with regard to ethical considerations was not on the agenda. People with disabilities came and then left for their respective sections. They had different types of accommodation; some were from a group residential home, a residential home with special services, and their own home or lived with their relatives. These people travelled to and from their daily activity centre using paratransit. The staff and residents shared the same areas in the sections. The premises appeared relatively neutral impression, apart from the so-called sensory rooms, where colours were used in order to stimulate cognitive activities. Each section had a living room with a kitchen and a number of smaller personal rest rooms. The sections’ kitchens were fully customized, e.g., doors opened automatically and the sink was height-adjustable. The sensory rooms were appreciated by the visitors, and there was quite a variety to choose from, for example, a white room (relaxing), a brightly coloured room (stimulating) and another room containing a rocking chair. The staff who showed us around reflected on the fact that “these rooms should be used more by staff, during breaks or after a work shift in order to increase their sense of well-being”. Also, the importance about greater awareness of ethical principles has been highlighted.

The staff’s areas of responsibility were documented in the implementation plan. These related to the support and service that each individual required. There was one file per person that contained the implementation plan and other documentation. The joint activities that took place in collective form were created and supervised by one of the staff members. This included, for example, baking together, visiting the nearby school to have coffee, as well as drama activities. The welfare technology available was sufficient for the planned activities although the building was equipped to cater for more advanced technical aids. The welfare technology that was available assisted the staff in carrying out support measures, but the staff pointed out that some technical equipment was not being fully utilized and that the technology was sometimes difficult to manage. One of the staff members stated “we are always bumping into each other because the handwritten notes are unclear about what we are supposed to be doing in the daily activities”. Communication via applications from work phones would “simplify our cooperation”, suggested another staff member—and ethical analysis was welcomed.

The staff indicated that the “welfare technology equipment that was in place, such as bed lifts, walkers and wheelchairs was very helpful to them in providing support, although a smart key system and self-flushing toilets would help them even more”. The staff also stated that some applications, such as alarms and cameras for monitoring, could help them with better supervision of autistic residents. The staff pointed out that “computers should be equipped with new programmes for documentation, scheduling and communication”, particularly regarding ethics.

Both management and staff were eager to gain increased competence within welfare technology. The aim was for more people in the staff group to be able to use advanced welfare technology associated with specific support measures. This was regarded as both an opportunity to increase knowledge among the staff and also a benefit in terms of the residents’ development. This awareness was evident during a workplace meeting concerning ethical analysis, with the manager and staff. The staff alluded to the importance of increased competence about the impact of ethical principles on future challenges. The staff emphasized that “increased knowledge about welfare technology was important for them, in order to avoid repeated decisions on delegations to the same person of the staff”. People with severe disabilities required the staff to be more knowledgeable about ethical principles. If the staff’s knowledge of ethical principles increased, it could contribute to improvements in security and confidentiality with regard to the documentation of information and communication, as well as stimulation in terms of the ethical benefits for the well-being of both staff and residents.

### Ethics and spaces

The participants’ answers in the questionnaire illustrates the importance of ethical analysis connected to the implementation welfare technology. Welfare technology was considered to be both frightening and fascinating. The participants mainly associated welfare technology with smart technical “tools” that facilitated everyday life. The responses suggested that questions of IT technology facilitate communication, learning, treatment and remote handling, control and information. The participants stated that welfare technology “facilitated their work” with reminders and scheduling during their working day, but that it could also function purely as an aid, for example, pressure-sensitive detection mats that could prevent fall injuries in people with disabilities. Welfare technology made their work “more efficient” through shorter and faster pathways to authority for processing and decision-making. It was also stated that welfare technology, to a certain extent, could “solve the shortage” of staff and the increasing needs of health and social care related to an ageing population. It was suggested that the development had happened rapidly and that a lot of responsibility was placed on the individual to familiarize themselves with practical usage by reading quick guides and learning finding out which rules apply.

The participants stated there was great “development potential” regarding welfare technology and they saw no ethical limitations on the continued development within the area. It was stated that welfare technology provided increased independence and autonomy, thereby improving well-being, as the technology reduced the dependence on others. Access to welfare technology was also something that was available to everyone. The participants stated that they often received “poor information” about how they were to practically manage welfare technology, which resulted in an increased burden of work and also ethical dilemmas. It was stated that older people, including people with disabilities, might find it difficult to book visiting times and search for information on the internet, or in situations where a mobile phone was required when paying for a ticket.

Welfare technology was described as a means of helping weak persons, but that it could also provide the opportunity for the strong persons to exercise power. The participants argued that the “benefit” of welfare technology could be seen from different perspectives: from the perspective of a person with disabilities, from the perspective of their loved ones and from the perspective of the staff. When welfare technology was implemented, it was important to ethically reflect on who would benefit from the technical solution, the individual with disabilities or the organization/staff. It was emphasized that welfare technology could never replace physical touch, conversation and human interaction. The participants stated that they were seeking “access to effective, safe, fast and health-promoting welfare technology” that took into account their personal integrity and well-being.

The participants stressed that it was important that a person with disabilities was involved when welfare technology was introduced. It was also stated that a person with disabilities had to give their clear consent and that no one could be forced to use new technology. It was suggested that welfare technology must be developed based on the needs of people with disabilities that they must be involved in the development and that welfare technology became a part of their everyday lives. It was stated that it was important that welfare technology reached out to everyone, regardless of their functional ability. Welfare technology would provide “added value” for a person with disabilities in the form of, for example, increased security and well-being. The participants also suggested that the use of welfare technology could entail risks for both people with disabilities and staff. It was stated that there was an ethical risk that the technology could become more important than the people with disabilities and that this could harm the social relationship with the person.

### Healthcare activities and ethical values

The focus group discussions underline the importance of ethical analysis to a better understanding of the implementation of welfare technology. Welfare technology was described as not always being based on the needs or wishes of either the staff or the person with disabilities. Thus, it could be perceived as an ethical obstacle in their everyday lives. The participants described that new technical solutions were introduced as it was considered to be of benefit to the work of the staff or to the person with disabilities, but without being involved in any ethical analysis in accordance with ethical principles before the decision was made to introduce the welfare technology.

“I can imagine that it will not be a matter of choice eventually, but rather, this is what we are offering”.

Welfare technology was introduced from different directions in the organization and was sometimes based on policy decisions. Participants considered it as being optional to use welfare technology in their daily work, but became uncertain in situations involving ethical problems when they were not aware of the basis for the policy decisions. It was stated that collaboration across boundaries, both within the social welfare administration as well as with neighbouring administrations, could increase the possibility of the introduction and use of welfare technology. There was, for example, a demand for increased responsiveness to people with disabilities, their relatives and the staff, and to their wishes and well-being. It was also stated that ethical problems sometimes arose when staff and for example, some person with disabilities had different opinions about which welfare technology was needed, and for whom it was needed. The principle of autonomy was not taken into account.

“Instead of just really seeing; this person really does not want this and because she wants independence, then it has to be like this and we have to learn to respect and accept it; that it is that person’s free will and it is this that can push the technology forward, that we can become more independent.”

The participants described some problems with the implementation of welfare technology and the decisions they were to make in terms of risks. An important aspect was that the staff did not know how to use certain types of welfare technology, which is the opposite of the ethical principle of doing no harm.

“I have never once been offered training in this and I have stated on several occasions that in the case of digital tools, if we’re going to use them, we need more training.”.

When the implementation of welfare technology became an accepted reality, certain welfare technology became part of working routines and the ethical discussions subsided. The participants also stated that there were a few problems with inertia in the organization and that there was a long interval between when they were informed about the introduction of new welfare technology and when it became implemented in operations, but that once welfare technology had been implemented, the process would be quick. They argued that it was important that caution was exercised when implementing welfare technology if such implementation was to become a lasting routine, because the process of implementation would be disassociated from ethical analysis.

“Having the staff involved in creating the programme, for example, using this self-checking programme that we’re starting with, where I have carefully presented that we are part of creating the content of the checks that we have and want to have. We can be involved and help improve the system beyond what was conceived from the start, so that we’re co-creators of the programme; this is probably not a bad idea”.

Welfare technology was seen as an aid that could let the staff spend more time with people with disabilities, which increased their well-being. It was also the case that welfare technology could be regarded as a tool that took over the staff’s tasks, thus making them redundant. This made the ethical discussion very important.

The participants want to utilize each other’s knowledge of welfare technology to a much greater extent and learn from each other within the administration. It was emphasized that they had problems finding time to learn about all the technology because they always worked under time constraints, which meant there was an ethical dilemma when there was no time left for skills development. The participants underlined the need for IT developers who work on reducing boundaries and barriers between different activities when it comes to welfare technology. The participants also discussed the opportunity of learning about welfare technology from each other. This exchange of knowledge would benefit people with disabilities so that the same digital solutions could be used more frequently, regardless of which facility the person was attached to. This is in line with the ethical principle of benefit.

“We need more time … yes, knowledge and resources, tools that are available quickly and easily should it be. Easily accessible knowledge.”

The participants explained that when implementing welfare technology, the goal should be for a person with disabilities to maintain and increase their capacity and opportunity to participate in activities in order to achieve a sense of well-being, independence and autonomy. The participants suggested that a person with disabilities should be able to own their records, which would be openly accessible material that relatives could read, depending on permission given by the individual. It would also make things easier if relatives were able to read certain entries in the records in order to see what a person with disabilities had been doing during the day

“It feels positive, you show to relatives what we do, what does Carl do together with us, and what does Carl do in the accommodation?”

The participants indicated that the level of knowledge regarding ethics and welfare technology varied between different organizations. They stated that new technology should be more easily accessible to everyone and should be based on the fair distribution of welfare technology in which all organizations have the same opportunities and progress equally in their development, in accordance with the ethical principle of justice. Regularly reflecting on ethical problems in staff groups was considered to be very important. New employees felt that they were unaware of the welfare technology that was available at other facilities of the administration. There was also a discussion on the prejudices that existed. They stated that both males and females had know-how and knowledge gaps when it came to using new welfare technology, regardless of ethical principles. It was more a matter of personal interest in the technology than a gender issue. They reflected on the age group distribution of the test groups and felt that age was not an obstacle to the development of welfare technology, but rather that welfare technology was also in demand among the elderly. The participants suggested that people with disabilities and their knowledge of welfare technology had increased, which required increased competence and staff development.

“I think it is very important to have ambassadors in the [staff] group who can also continuously keep up to speed and pursue this, so that this becomes their task, and so that there is someone taking care of it who can deal with the issues that arise when I’m not there”.

## Discussion

It is important to note that the organizations have responsibility for producing assessments that are as useful as possible to their users before implementing welfare technology, and which ultimately benefit public health and well-being. Welfare technology is never free of ethical values. So, making these values explicit is key to increasing international transferability and policy relevance. When decisions are made to implement welfare technology, it requires resources to be allocated. Choosing one technology may involve the devaluation or replacement of another technology, and also lead to the reallocation of resources within health care or between wider sectors of society. Ideally, policymakers are expected to balance individual and broader societal interests, taking into consideration all the ethical values at stake.

This study underlines that the impact of ethical analysis connected to the implementation of welfare technology in social services provide insight and assist the improvement of the decision-makers in interpreting information in a relevant way in terms of developing policy. An ethical analysis can be said to be a strategy and tool for assessing trade-offs when there are conflicts between different interests or values (The Swedish National Council of Medical Ethics, 2018). Thus, by conducting an ethical analysis, it is possible to establish a position on issues where there are different values and conflicts of interest. It is therefore important to include risk analyses for processes involved in the implementation of new welfare technology, with the goal of ensuring they are ethically appropriate. This indicates that the use of ethical analysis would contribute to a more accurate and systematic approach when the implementation of new welfare technology improves areas of social services within a municipality. It would help to ensure a better understanding and utilization of welfare technology, particularly in terms of transparency for all parties involved, and for greater awareness of ethical principles associated with the implementation of welfare technology.

When welfare technology is to be introduced within organizations (in this study, within the framework of social services in a municipality), a working model using an ethical analysis could contribute valuable data to decision-making regarding different interventions for people with disabilities. This ethical awareness becomes a tool for assessing trade-offs when there are conflicts between different interests or values in order to strengthen the well-being of people with disabilities within the context of health and social care. However, these conflicts are not obvious.

The implementation of welfare technology has to take into account key ethical principles, “autonomy, benefit, doing no harm, fairness”, in issues in which there are different values and conflicts of interest. This is important in order to ensure that decisions regarding the introduction and implementation of welfare technology are not perceived as an obstacle but rather as a stimulus, in terms of both increased knowledge and competence and in terms of well-being (regardless of gender, age, origin, variations, etc.), in the innovation development that is currently taking place within health and social care. Andersson Marchesoni et al. (2012) suggests that when staff members form an opinion on welfare technology, they often base this on their past experience. Many people keep their distance and feel it does not concern them or they cannot see the benefits of welfare technology, because their ethical reflections are in the background. Employees sometimes feel forced by the organization to start working with the welfare technology that has been implemented, while others see it as a healthcare development and become inspired (Andersson Marchesoni et al., 2012). For the staff in this study, it was important to be able to focus on a person with disabilities as being unique, which the staff believed could be compromised by time-consuming welfare technology—based on the ethical analysis. The staff wanted to feel that they had the skills to use welfare technology, which is also confirmed by Andersson Marchesoni et al. (2017).

The successful implementation of new welfare technology in a municipality requires reliable infrastructure and a fully prepared organization. In addition, staff competence needs to increase with regard to managing the various challenges of welfare technology (Andersson et al., 2016) related to discussions on ethical analysis. The staff in this study emphasized the importance of the resources to be allocated when implementing welfare technology, which is confirmed in a previous study (Rundkvist, 2018). If collaboration improves, the exchange of knowledge increases and experience is utilized, which can lead to staff feeling valued and inspired to find more innovative solutions to problems that arise. There is a need for the innovative ideas of staff to be heard regarding welfare technology solutions, which could contribute to both improving and facilitating their work, as well as the everyday life of people with disabilities, in accordance with ethical principles. A more innovative culture, capable of gathering ideas from the staff, for example, through ambassadors and networks, would help the staff become more engaged and would result in more innovations. The results of this study were confirmed by Denti and Hemlin (2012), who emphasized that conscious management was the success factor that increased an organization’s innovative potential. A characteristic of leadership that seizes on innovations is that it stimulates the internal driving forces of its staff when it comes to developing new innovations. It is also about identifying staff who possess knowledge and skills and getting them involved in various innovation processes. Culture carriers and inspirers are needed in order to successfully disseminate a municipality’s digital vision (Denti & Hemlin, 2012).

The staff in this study welcomed welfare technology. Thus, it was of great importance that the staff were continuously given the opportunity to develop their skills within the area regarding, for example, safety aspects, in order to be able to keep up with the rapid developments and be able to support people with disabilities in using welfare technology, in accordance with ethical principles. There are indications that the staff are concerned that people with disabilities will have less human contact with the staff if welfare technology is implemented, but also that the autonomy of people with disabilities would decrease (Zwijsen et al., 2011). This could be one reason why the staff in this study did not use welfare technology to its full capacity, although it also emerged that they lacked the knowledge and skills required to use welfare technology. An important challenge in implementing welfare technology is to provide ethical analyses, sufficient support and staff training until the use of welfare technology becomes a routine in everyday activities.

The staff in this study indicated that it was important for welfare technology to be developed based on the needs and preferences of people with disabilities, and that it could be used regardless of the activity the individual was engaged in during the course of the day, something that was also alluded to by Peek et al. (2014). People with disabilities have a right to participation and autonomy in their everyday life and the staff believed that it was important that welfare technology increased this possibility through greater participation when assessing assistance needs and in conjunction with the introduction of new digital services and products, based on ethical analysis. One challenge was that welfare technology needed to be secure and had to be easy to understand and manage in order to give the user more confidence in its use. Boström et al. (2013) described the importance of people with disabilities also having the time and opportunity to get used to and learn about welfare technology which, in turn, makes it easier to accept it. If welfare technology promotes the integrity and autonomy of people with disabilities, this can provide a sense of security (Boström et al., 2013). Thus, decisions about who is allocated support and how implementation of welfare technology in the organization is carried out shall be based on ethical principles.

Ethical discussions may arise concerning fairness, integrity and the importance of people with disabilities not being excluded from the rest of society. However, it is also essential that certain areas and activities are not prioritized and allocated disproportionate amount of welfare technology. In accordance with the Social Services Act, the goal of social services is to promote people’s economic and social security, equality of living conditions and active participation in the life of the community (SFS 2001:453, 2001, §1). In order to achieve these goals, it is important that staff receive support and guidance in the ethical dilemmas that arise, but also that clear steering documents and guidelines are drawn up. It is important to gain an understanding that staff have different opinions about technology and that it must not be taken for granted that gender or age are decisive factors for the competence of people with disabilities or staff when new digital services are introduced. Thus, in connection with staff training initiatives, it is important to integrate a gender and norm-critical perspective. It is also important to gain a good picture of how a potential conflict situation would appear when implementing welfare technology, in accordance with ethics. By examining which stakeholders are involved, it becomes easier to identify and divide the problems and ethical dilemmas into a situation in which well-being is the focus. Different stakeholders do not perceive and describe the situation in the same way. Particularly important stakeholders within the sphere of social welfare administration include people with disabilities, their relatives, staff, politicians, officials at different levels and taxpayers. As a next step, it would be useful to clarify which ethical values and/or interests are at stake among stakeholders. Based on the information provided, different options for actions can be crystallized. These can be based, for example, on the needs of people with disabilities, laws, technical possibilities, access to staff and how different actors have acted in the past. There then needs to be an investigation into whether there is any conflict of interest in the situation that has arisen, as well as a need for greater awareness about ethics associated with the implementation of welfare technology.

## Conclusions

This study was about the awareness of the need of ethical analysis, in spaces in which welfare technology is implemented, particularly within areas of social services in a Swedish municipality. The findings indicated that improving ethical analysis, when the implementation of new welfare technology is included in daily activities within areas of social services, contributed to a more accurate and systemic approach. It will help to ensure a better understanding and use of welfare technology, particularly in terms of clarity and transparency for all the involved parties. Welfare technology was welcomed by the staff, who usually contributed with constructive ethical reflections based on their experiences.

The successful implementation of new welfare technology in a municipality requires ethical analysis, reliable infrastructure and a fully prepared organization. Staff competence also needs to increase with regard to handling the various challenges of welfare technology related to ethical principles. It is of great importance that staff continuously are given the opportunity to develop their skills in order to be able to keep up a rapid development in using welfare technology based on ethical analysis, to be able to support people with disabilities. People with disabilities have a right to participation and autonomy in their everyday lives, and the staff believed that welfare technology increased this possibility. Thus, decisions about who is allocated support and how the implementation of welfare technology in the organization is carried out shall be based on ethical principles. In other words, ethics associated with rules and implementation, which benefit their well-being.
